# Activin B-activated Cdc42 signaling plays a key role in regulating adipose-derived mesenchymal stem cells-mediated skin wound healing

**DOI:** 10.1186/s13287-022-02918-9

**Published:** 2022-06-11

**Authors:** Simin Huang, Xueer Wang, Min Zhang, Mianbo Huang, Yuan Yan, Yinghua Chen, Yijia Zhang, Jinfu Xu, Lingwei Bu, Ruyi Fan, Huiyi Tang, Canjun Zeng, Lu Zhang, Lin Zhang

**Affiliations:** 1 Department of Histology and Embryology, NMPA Key Laboratory for Safety Evaluation of Cosmetics, Key Laboratory of Construction and Detection in Tissue Engineering of Guangdong Province, School of Basic Medical Sciences, Southern Medical University, Guangzhou, 510515 People’s Republic of China; 2 Key Laboratory of Functional Proteomics of Guangdong Province, Key Laboratory of Mental Health of the Ministry of Education, School of Basic Medical Sciences, Southern Medical University, Guangzhou, 510515 China; 3 Department of Orthopedics, Third Affiliated Hospital of Southern Medical University, Academy of Orthopedics Guangdong Province, Guangzhou, 510630 Guangdong China

**Keywords:** ADSCs, Wound healing, Cdc42, RNA-seq

## Abstract

**Background:**

In our previous study, activin B in combination with ADSCs enhances skin wound healing. However, the underlying molecular mechanisms are not well studied. Cdc42 is recognized to play a critical role in the regulation of stem cells.

**Methods:**

Pull-down assay was performed to investigate the activity of Cdc42. The dominant-negative mutant of Cdc42 (Cdc42N17) was used to explore the role of Cdc42 in activin B-induced ADSCs migration, proliferation, and secretion in vitro*.* Cdc42N17-transfected ADSCs were injected into a full-thickness excisional wound model to explore their efficiency in wound healing in vivo. The wound healing efficacy was evaluated by the wound closure rates and histological examination. The neovascularization and wound contraction were detected by immunohistochemistry staining of CD31 and α-SMA. Finally, the underlying mechanisms were explored by RNA sequencing.

**Results:**

Cdc42N17 inhibited ADSCs migration, proliferation, and secretion induced by activin B. Furthermore, Cdc42N17-transfected ADSCs inhibited the wound closure rate and suppressed the expression of CD31 and α-SMA induced by activin B in vivo*.* The RNA sequencing showed that the differentially expressed genes in Cdc42N17-transfected ADSCs versus ADSCs were associated with cell migration, proliferation, and adhesion. Further study revealed that the Cdc42-Erk-Srf pathway was required for activin B-induced proliferation in ADSCs.

**Conclusions:**

Our study indicates that Cdc42 plays a crucial role in ADSCs-mediated skin wound healing induced by activin B.

**Graphical Abstract:**

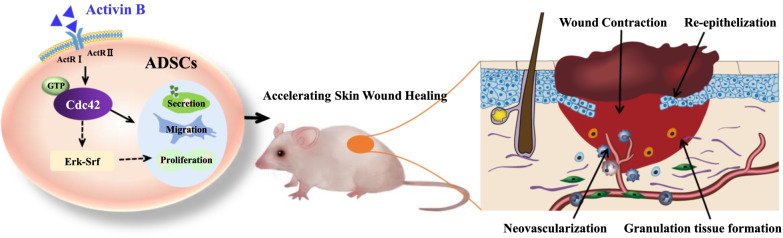

**Supplementary Information:**

The online version contains supplementary material available at 10.1186/s13287-022-02918-9.

## Introduction

Stem-cell-based therapeutic strategies have shown considerable potential to improve the rate and quality of skin wound healing [1, 2]. Among a wide variety of stem cells that show efficacies in wound healing, adipose-derived mesenchymal stem cells (ADSCs) seem to possess the least limitations for clinical applications [3, 4]. Transplantation of ADSCs into full-thickness wounds is able to improve wound healing by promoting angiogenesis, modulating immune response, and inducing epithelialization in the wound [5, 6].

Many growth factors and cytokines were shown to play a key role in the process of cutaneous wound healing [7–10]. For example, activin B, a member of the TGF-β superfamily, is involved in the process of skin wound repair [11, 12]. In a previous study, we reported that activin B was able to induce actin stress fiber formation and migration of ADSCs to promote skin wound healing [13]. However, the mechanism in this process is still poorly understood.

Cell division cycle 42 (Cdc42), a member of the Rho GTPases family, contributes to cell migration by controlling protrusions, adhesion, and contraction [14–17]. Cdc42 is crucial for the regulation of stem cells’ behaviors. Loss of Cdc42 reduces α-SMA expression in mesenchymal stem cells [18]. The elevated activity of the Cdc42 in aged hematopoietic stem cells (HSCs) correlates with a loss of polarity [19]. In a recent study, we have found that Cdc42 influences the morphology and skeleton in bone marrow-derived mesenchymal stem cells (BMSCs) [20]. However, the role of Cdc42 in ADSCs-mediated wound healing remains unclear.

In this study, we used the dominant-negative mutant of Cdc42 (Cdc42N17) to explore the role of Cdc42 in activin B-induced ADSCs migration, proliferation, and secretion in vitro. Cdc42N17-transfected ADSCs were injected into a full-thickness excisional wound model to explore their efficiency in wound healing in vivo. Moreover, RNA sequencing was used to explore the downstream mechanism of Cdc42 in the regulation of wound healing processes. Our study reveals an essential role of Cdc42 in ADSCs-mediated wound healing.

## Material and methods

### Animals

One hundred and eight specific pathogen-free (SPF) class C57BL/6 male mice (26–28 g) of 3 months old were purchased from Laboratory Animal Center of Southern Medical University (Guangzhou, China; SCXK 2021-0041). The mice were housed in a temperature-controlled room (23 ± 2 °C) under a 12-h light/dark cycle with available food and water ad libitum. The mice were allowed 1 week to adapt to the environment before experiments. All the procedures related to the care and use of animals in this study were in accordance with the guidelines of National Institutes of Health (NIH) and was approved by the Bioethics Committee of Southern Medical University (Approval number: L2019084).

### Isolation, identification, and labeling of ADSCs

ADSCs’ isolation was performed as previously reported [21, 22]. Mice were killed by cervical dislocation and asepticized by soaking in 75% ethanol for 5 min. The inguinal adipose tissues were withdrawn and washed with PBS at 4 °C for three times. After removing the blood vessels and lymph nodes carefully, the adipose was cut into pieces and digested for 90 min at 37 °C using 10% fetal bovine serum (FBS) and 125 U/mL collagenase (Cat# 17018029, Collagenase Type I, Gibco) [23]. By filtrating through 100-μm nylon filter mesh (Cat# 352360, BD Falcon) and concentrated by centrifugation at 300*g* for 5 min, the stromal vascular fraction (SVF) was attained [24]. Then, ADSCs were resuspended in complete medium (high-glucose Dulbecco’s modified Eagle’s medium (H-DMEM) with 10% FBS and 1% penicillin-streptomycin solution (Cat# C0222, Beyotime, China)) and cultured in a humid atmosphere with 5% CO_2_ at 37 °C. The culture medium was updated every 48 h. The cells were monitored daily under an inverted phase-contrast microscope (Leica DMI4000 B) and passaged when they reached 80–90% confluence.

Cells at passage three were used for 5,6-carboxyfluorescein diacetate succinimidyl ester (CFSE) (Cat# C1157, Invitrogen) labeling and identification according to the standard listed in Additional file 1.

### Lentivirus infection

The dominant negative mutant Cdc42N17, the constitutively active mutant Cdc42L61, and the EGFP lentiviral vector were generated as described in our previous study [20, 22]. The titers of Cdc42N17, Cdc42L61, and EGFP vector were 1.63 × 10^9^ TU/ml, 6.93 × 10^8^ TU/ml, and 4.53 × 10^8^ TU/ml, respectively. The lentivirus was serum-free incubated with ADSCs for 8 h. On the day 3 after lentivirus infection, the infection efficiency was determined by examining green fluorescence in the cells using fluorescent microscopy and pull-down assay.

### The inhibitor treatment

Cells were serum-starved for 12 h and then treated with serum-free H-DMEM containing Srf inhibitor CCG-100602 (15 μM, Cat# HY-120855, Mce) for 24 h [25, 26] or Erk1/2 inhibitor SCH772984 (5 μM, Cat# S7101, Selleck) for 2 h [27].

### GST pull-down assay

GTPase pull-down assay was performed according to the manufacturer's protocol (1:100, Cat # 14-325, Rac/cdc42 Assay Reagent, Millipore) as described previously [20, 28]. In short, after lysed with MLB buffer and determined the total protein concentration, the cell lysates were divided into two equal parts: One was blotted for total Cdc42, and the other was subjected to a PAK1 PBD binding assay. The beads with Cdc42 GTP-bound were captured, and the activation levels of Cdc42 were analyzed by western blot using an anti-Cdc42 antibody. The detailed protocol for pull-down assay is described in Additional file 1.

### Western blot analysis

Cells were lysed using radio-immunoprecipitation assay (RIPA) buffer (Cat# P0013B, Beyotime, China), and total protein was extracted and normalized according to the concentrations determined by Enhanced BCA Protein Assay Kit (Cat# P0010, Beyotime, China). The total protein was then re-suspended with 5 × loading buffer (Ca# P0286, Beyotime, China) and boiled for 5 min. Twenty-five mg protein from each sample was separated by electrophoresis on 10% polyacrylamide gels (Cat# PG112, Epizyme, China) and transferred onto polyvinylidene difluoride membranes. The blots were blocked with 5% skim milk (Cat# 232100, Genebase, China) and incubated with a primary antibody Cdc42 (1:2000, Cat# ab187643, Abcam), Srf (1:1000, Cat# 16821-1-AP, Proteintech, China), or phosphorylation Erk (p-Erk) (1:1000, Cat# 4370S, Cell Signaling) at 4 °C overnight. After further incubated with HRP-conjugated anti-rabbit IgG (1:2000, Cat# ab6721, Abcam) secondary antibody for 1 h at room temperature, the bands’ intensity was visualized using the enhanced chemiluminescence (ECL) luminescence reagent (Cat# MA0186-1, Meilunbio, China) and Gel Image System (Tanon-5200CE). The blots were then washed with a commercial stripping buffer (Cat# P0025N, Beyotime, China) for 10 min at room temperature and re-probed with antibodies against total Erk (1:1000, Cat# 4695S, Cell Signaling). Then, the intensity of the band was quantified using ECL and Gel Image System (Tanon-5200CE).

### Migration assay

ADSCs and cells transduced with Cdc42N17, Cdc42L61, or EGFP vector were seeded into 24-well plastic plates at the density of 1 × 10^5^ cells per well and cultured until they reached a confluence of 90%. After serum-starved for 12 h, the cells were divided into seven groups. Among them, four groups (ADSCs, Cdc42N17, Cdc42L61, and EGFP vector) were treated with serum-free H-DMEM, while the other three groups (activin B+ADSCs, activin B+Cdc42N17, and activin B+Cdc42L61) were treated with H-DMEM containing 10 ng/ml activin B. Then, the migration properties of cells were analyzed by the following two methods.

For the scratch wound healing assay, the uniform scratch wounds were scraped and photographed at 0, 24, and 48 h after being washed with PBS. The Image-Pro Plus 6.0 software was used to calculate the scratching area.

For the transwell assay, 500 µl preheated serum-free H-DMEM was added to the upper and lower chamber, respectively, and incubated for 1 h at 37 °C. After discarding the medium, a total of 200 μl serum-free media including 5 × 10^4^ cells has been added into the upper chamber, while the lower chamber was filled with 300 µl different culture media (H-DMEM or H-DMEM containing 10 ng/ml activin B) as designed previously. After 24-h incubation, the migrated cells under the chamber were fixed with 4% paraformaldehyde solution (PFA) (Cat# G0528, GBCBIO, China) for 15 min, stained with crystal violet staining solution (Cat# C0121, Beyotime, China) and imagined by microscope.

### Proliferation assay

For the cell proliferation experiment, ADSCs and cells transduced with Cdc42N17, Cdc42L61, or the mock vector were seeded on cover glass into 24-well plastic plates at the density of 1 × 10^5^ cells per well. After serum-starved for 12 h, the cells were divided into eleven groups. Among them, six groups (ADSCs, Cdc42N17, Cdc42L61, CCG-100602, SCH772984, and EGFP vector) were treated with serum-free H-DMEM, while the other five groups (activin B+ADSCs, activin B+Cdc42N17, activin B+Cdc42L61, activin B+CCG-100602, and activin B+SCH772984) were treated with H-DMEM containing 10 ng/ml activin B. Then, an addition of 12.5 μM EdU was added in each group. After incubating for 6 h at 37 °C, cells were fixing, Apollo- and nuclear-stained as manufacturer’s instructions (Cat# C10310-1, RiboBio, China). The EdU-positive cells were observed under fluorescence microscope.

### Cytokine analysis

The cells of ADSCs and cells transduced with Cdc42N17 or Cdc42L61 have been selected for a 12-h serum-free starve. After incubating for 24 h with H-DMEM (for ADSCs, Cdc42N17, and Cdc42L61) or H-DMEM with 10 ng/ml activin B (for activin B+ADSCs, activin B+Cdc42N17, and activin B+Cdc42L61), the cell culture supernatant was collected by centrifuging at 3000*g* for 15 min and filtering with a 0.22-μm filter to remove apoptotic cells and debris. Then, the secretion levels of Col 1 (Cat# MU30364, Bisowamp, China) and VEGF (Cat# MU30236, Bisowamp, China) were detected using a commercially available enzyme-linked immunosorbent assay (ELISA) system following the kit’s instructions.

### Wound healing model

The wound healing model was established as we described previously [13]. Mice were anesthetized by injecting 2% pentobarbital sodium (1.5 mL/kg) into their intraperitoneal. After the dorsal hair was depilated using a honey and wax mixture and cleaned with 70% ethanol, skin punch was used to delineate an 8-mm round-shaped image on the skin. Then, sterile scissors were applied to create full-thickness skin wounds on both the right and left shaved dorsal skin after povidone iodine disinfection. Each mouse had retained the capability to free ingest food and water after the operation.

After wounding, mice were randomly divided into six groups (*n* = 6 for each group) according to the treatment on the site surrounding the wound (Table 1) daily for 3 days. The cells and/or activin B were resuspended in PBS and administered to the wounds by subcutaneous injection. Wound areas were measured photographically at days 0, 3, 7, and 14 after surgery, and the rate of wound closure was calculated with the following equation: wound closure rate (%) = [(original wound area − open area on final day)/original wound area] × 100%.

**Table 1:** Group on wound healing model

Group	Name	Treatment
1	Control	0.4 ml PBS
2	Act B	0.4 ml 10 ng/ml activin B
3	ADSCs	0.4 ml 6 × 10^6^/ml ADSCs
4	Act B+ADSCs	0.4 ml 6 × 10^6^/ml ADSCs induced by 10 ng/ml activin B for 12 h
5	ADSCs (Cdc42N17)	0.4 ml 6 × 10^6^/ml ADSCs transduced with Cdc42N17
6	Act B+ADSCs (Cdc42N17)	0.4 ml 6 × 10^6^/ml ADSCs transduced with Cdc42N17 and induced by 10 ng/ml activin B for 12 h

### Frozen sections

For analysis of the applied ADSCs within the wound site, the wound and surrounding tissue were collected on day 3 and frozen with optimal cutting temperature (OCT) compound. Serial sections (15 μm) were taken from the wound center to the edge by a freezing microtome at − 20 °C and adhered to the slides. After washing with PBS and blocking in PBS containing 5% bovine serum albumin (BSA) (Cat# ST023, Beyotime, China), the sections were stained with Hoechst 33258 (1:500, Cat# H21491, Invitrogen) for 10 min and examined by fluorescence microscopy.

### Hematoxylin and eosin (H&E) staining and histological evaluation

Mice were killed at postoperative 3rd, 7th, and 14th day, respectively. Then, complete wound with 0.5-cm margin was carefully cut down and rinsed with PBS before and after fixing by 4% paraformaldehyde solution (PFA) (Cat# G0528, GBCBIO, China) for 3 days. Then, the samples were dehydrated and embedded in paraffin. Serial sections (5 µm) were collected and H&E-stained according to the detailed method described in Additional file 1.

For histological evaluation, re-epithelialization and granulation tissue formation was evaluating by using H&E staining. The length of epithelial tongues of wound skin sections on each group sample (*n* = 6) was measured between the wound edge and the leading edge of migrating epithelial tongue with ImageJ 1.52a software (National Institutes of Health, Bethesda, MD, USA). For the granulation tissue formation, each group sample (*n* = 6) was given a granulation tissue score ranging from 1 to 12. The criteria used for granulation tissue scores of wound healing were referred to previous researches [29–32] and our previous study [33] and summarized in Additional file 1: Table S1.

### Masson’s trichrome staining

The slices adjacent to the wound center were used for Masson’s trichrome staining using Masson’s trichrome staining kit (Cat# MST-8004, Maixin-Bio, China) according to the manufacturer’s instructions.

### Immunohistochemical assay

The tissue slices were deparaffinized and rehydrated (detailed procedure as previously described in H&E staining). In order to retrieve antigen, samples were first immersed in 0.1 M citrate buffer at 96 °C for 10 min and later incubated in 5% BSA for 2 h. The slices were incubated with antibody against CD31 (1:100, Cat# ab281583, Abcam) and α-SMA (1:200, Cat# ab32575, Abcam) at 4 °C overnight. After rinsing with PBS and incubated with biotinylated goat anti-rabbit secondary antibody (Cat# PV-6001, ZSGB-BIO, China) for 2 h, the tissue slices were colored with 3,3-diaminobenzidine (DAB) (Cat# AR1022, Boster, China), stained with hematoxylin, dehydrated with a gradient ethanol series, soaked with xylene, and then sealed with resin. Finally, five random locations of each tissue slice were selected to count the new capillaries with endothelial cells positive for CD31 via microscope (400 ×) or to analyze the average optical density values for α-SMA expression at the indicated time points using Image-Pro Plus 6.0 Software.

### Total RNA preparation

ADSCs transduced with or without Cdc42N17 were seeded in 60-mm plastic culture plates. After 12-h serum-free starve, they were divided into four groups according to different processing methods with H-DMEM (ADSCs, Cdc42N17) or H-DMEM with 10 ng/ml activin B (Act B+ADSCs and Act B+Cdc42N17) and incubated for another 24 h. Total RNA of those cells was extracted, and genomic DNA was simultaneously eliminated using EZ-10 DNAaway RNA Mini-Preps Kit (Cat# B618133, Sangon Biotech, China) according to the manufacturer’s instructions. The quality and purity of the resulting RNA were checked using the NanoDrop™ spectrophotometer; only high-quality RNA sample (OD_260/280_ = 1.8–2.2, OD_260/230_ ≥ 2.0) could be used for sequencing and qPCR analysis.

### RNA sequencing

For the transcriptomic analysis, 1 μg total RNA was used to prepare RNA-seq transcriptome library using TruSeq™ RNA sample preparation kit from Illumina (San Diego, CA). The raw paired-end reads were trimmed, and the quality was controlled by SeqPrep and Sickle with default parameters. The high-quality clean data were aligned to the mouse genome using HISAT2 software [34], and mapped data (reads) of each sample were assembled by StringTie in a reference-based approach [35].

To identify differential expression genes (DEGs) between these samples, the expression levels of each transcript and gene abundance were calculated according to the transcripts per million reads (TPM) method and RSEM, respectively [36]. Essentially, differential expression analysis was performed using the DESeq2 [37]/EdgeR [38] with *Q* value ≤ 0.05; DEGs with |log2FC|> 1 and *Q* value ≤ 0.05 (DESeq2 or EdgeR)/*Q* value ≤ 0.001 (DEGseq) were considered to be significantly differentially expressed genes. In addition, functional enrichment analysis including GO and KEGG was performed to identify which DEGs were significantly enriched in GO terms and metabolic pathways at Bonferroni-corrected *P* value ≤ 0.05 compared with the whole-transcriptome background. GO functional enrichment and KEGG pathway analysis were carried out by Goatools and KOBAS [39].

### qPCR analysis

To further verify RNA-seq data, the genes described in Table S2 with significant expression differences were analyzed by qPCR using the Maxima SYBR Green/ROX qPCR Master Mix kit (Cat# K0221, Thermo Scientific) according to the manufacturer’s instructions. To be specific, 1.0 μg total RNA was converted to cDNA using MonScript™ RTIII All-in-One Mix with dsDNase kit (Cat# MR05101S, Monad Biotech, China), and the qPCR reactions were performed on the ABI 7900 system using the following program: 95 °C for 10 min, and 40 cycles of 95 °C for 10 s, 60 °C for 10 s, and 72 °C for 30 s, followed by dissociation steps. Reactions were run in triplicates. The *GAPDH* housekeeping gene was used as an internal control for data normalization across samples. The normalization of Ct values for each gene and the determination of fold changes in gene expression (normalized to control group) were calculated using the 2^−ΔΔCt^ method.

### Statistical analysis

Numerical data were expressed as the means ± standard deviation (SD). All experiments were performed at least three times. Statistical differences between the groups were assessed using one-way analysis of variance (ANOVA) in SPSS20.0 software (IBM, Armonk, NY, USA). A *p* value of less than 0.05 (*P < 0.05) was considered to be statistically significant.

## Results

### Characterization of isolated ADSCs

To validate ADSCs isolated from mice, we assessed cell surface markers using immunocytochemistry. In support of previous reports [13, 40], ADSCs were positive for CD44 and CD90, while nearly negative for CD31 and CD80 (Additional file 1: Fig. S1A). The flow cytometry was also assessed to characterize isolated ADSCs cell surface markers. ADSCs were positive for CD29, CD44, CD90.2 and nearly negative for CD31, CD45, and CD117 (Additional file 1: Fig. S1B). ADSCs were further characterized by confirming their ability to undergo specific osteogenic, adipogenic, and chondrogenic differentiation. These cells were positive for Alizarin Red staining (Additional file 1: Fig. S1C), oil red O staining (Additional file 1: Fig. S1D), and Alcian Blue staining (Additional file 1: Fig. S1E), indicating osteogenic, adipogenic, and chondrogenic respective cell-type differentiation. Only cells that met these criteria were used in subsequent experiments.

### Cdc42 regulates activin B-induced ADSCs migration

**Fig. 1:**
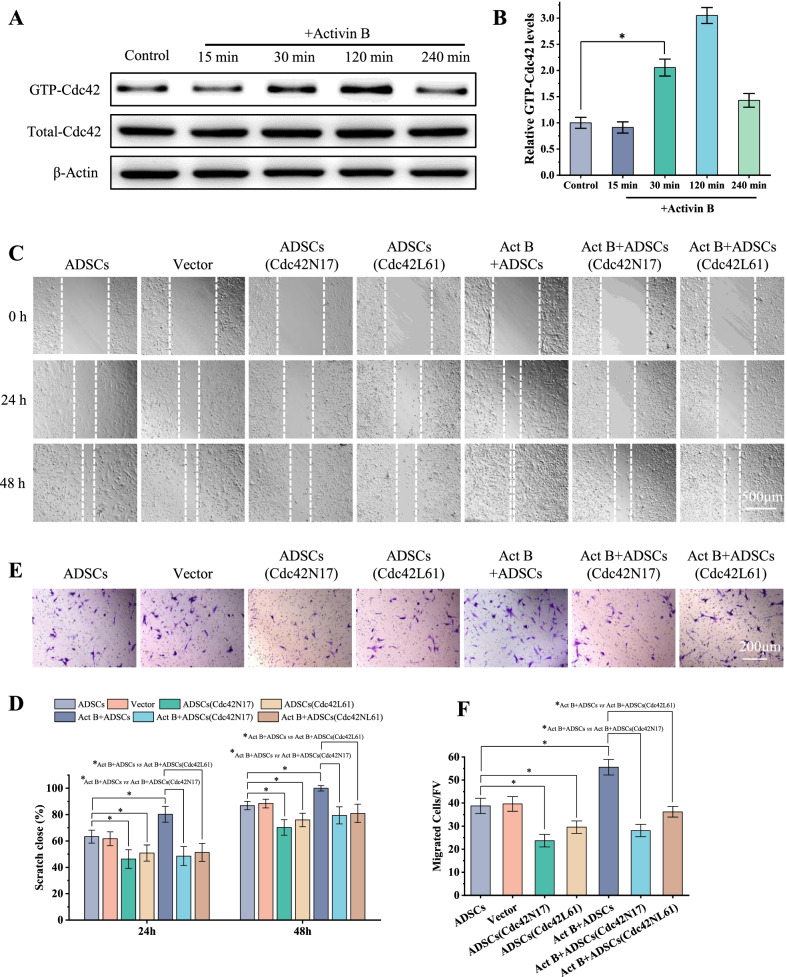
Cdc42 regulates activin B-mediated ADSCs migration in vitro. **A** Pull-down assays were performed to detect the amounts of GTP-bound Cdc42 in ADSCs, ADSCs treated with activin B, respectively. **B** The ratios of GTP-Cdc42 versus total Cdc42 levels were analyzed. **C** Scratch wound healing assays were performed to detect the migration of cells treated by seven methods, and photographs were taken at 0, 24, and 48 h after scratch injury. Scale bar = 500 μm. **D** The healing rates were quantified by measuring the area of the injured region. **E** Transwell assays were performed to detect the invasion of these cells, and photographs were taken at 24 h after seeds. Scale bar = 200 μm. **F** The migrated cells were counted. All values are expressed as mean ± SD from three independent repeats. *P < 0.05

We then investigated whether activin B regulated the activity of Cdc42 in ADSCs by GST pull-down assay. We found that the GTP-bound Cdc42 was increased from 30 to 120 min after activin B treatment (Fig. 1A, B).

To study the role of Cdc42 in activin B-induced ADSCs migration and other biological functions, the mock vector or the dominant-negative mutant of Cdc42 (Cdc42N17) and the constitutively active mutant of Cdc42 (Cdc42L61) were transduced into ADSCs, respectively. The efficient transduction of Cdc42N17 and Cdc42L61 was confirmed by GFP imaging and pull-down assay (Additional file 1: Fig. S2).

We next examined whether Cdc42 was involved in activin B-induced ADSCs migration. In a scratching assay, as reported previously [13], 10 ng/ml activin B effectively induced ADSCs migration (Fig. 1C, D). However, Cdc42N17 inhibited ADSCs migration with or without activin B after 24 and 48 h (Fig. 1C, D). The wound healing rate of activin B-stimulated Cdc42N17-transduced ADSCs was comparable to that of Cdc42N17-transduced ADSCs without activin B stimulation (Fig. 1C, D). These findings suggest that Cdc42N17 abolished activin B-induced ADSCs migration. Consistent with this finding, Cdc42N17 inhibited activin B-induced ADSCs migration to the lower chamber in a transwell assay (Fig. 1E, F). However, Cdc42L61 did not promote ADSCs migration with or without activin B (Fig. 1C–F). In summary, these results suggest that Cdc42 is necessary but not sufficient for activin B-induced ADSCs migration.

### Cdc42 regulates activin B-induced ADSCs proliferation and secretion

Next, we investigated the role of Cdc42 in the proliferation and secretion of ADSCs treated with or without activin B.

**Fig. 2:**
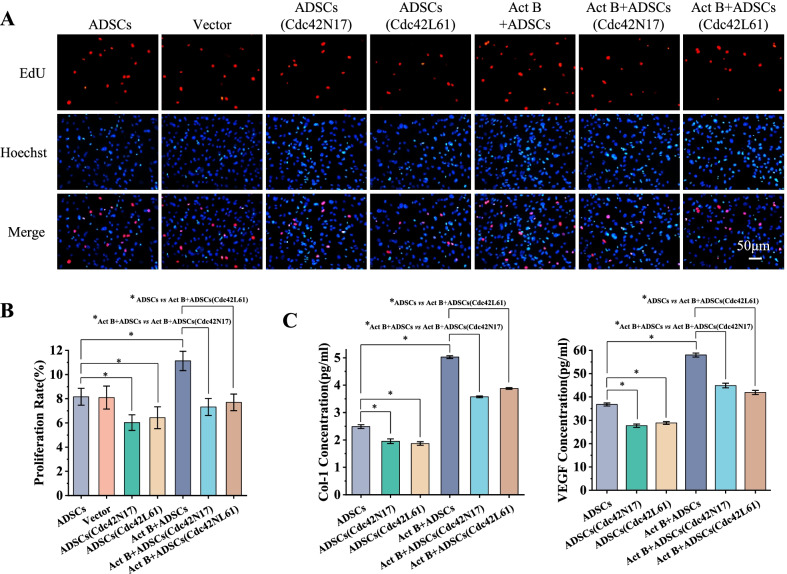
Cdc42 regulates activin B-mediated ADSCs’ proliferation and secretion in vitro. **A** Representative fluorescence imaging of EdU staining of seven groups cells at 6 h. Scale bar = 50 μm. **B** The proliferation rates were quantified by calculating the percentage of EdU-positive cells. **C** Average concentration of Col 1 and VEGF in the conditioned cell medium of six groups. All values are expressed as mean ± SD from three independent repeats. *P < 0.05

EdU-stained cell number was markedly increased in the activin B group compared with the control group. Cdc42N17 significantly decreased EdU-stained cell number in the ADSCs treated with or without activin B. Of note, EdU-stained cell number in activin B-stimulated Cdc42N17-transduced ADSCs was comparable to that in Cdc42N17-transduced ADSCs without activin B stimulation (Fig. 2A, B). These findings reveal that Cdc42 may regulate ADSCs’ proliferation induced by activin B. Moreover, the concentrations of Col 1 and VEGF were markedly increased in the activin B group compared with that in the control group, whereas Cdc42N17 significantly inhibited the secretion of Col 1 and VEGF in the ADSCs with or without activin B. Thus, Cdc42 contributes to ADSCs’ secretion induced by activin B (Fig. 2C). However, Cdc42L61 did not promote ADSCs’ proliferation and secretion with/without activin B (Fig. 2A-C). Taken together, these findings suggest that Cdc42 is necessary but not sufficient for ADSCs’ proliferation and secretion induced by activin B.

### Cdc42 promotes activin B-induced ADSCs-mediated skin wound healing in vivo

We then examined the role of Cdc42 on activin B-induced ADSCs-mediated cutaneous wound healing in vivo.

To assess ADSCs administered, 5, 6-carboxyfluorescein diacetate succinimidyl ester (CFSE) was used to label ADSCs before transplantation (Additional file 1: Fig. S3A) and then we collected the frozen sections on day 3 after ADSCs transplantation. Green fluorescence (EGFP+) cells were detected in the ADSCs group, activin B+ADSCs group, ADSCs (Cdc42N17) group, and activin B+ADSCs (Cdc42N17) group but not in the activin B group or control group (Additional file 1: Fig. S3B). These data suggest that ADSCs were incorporated into wound sites on day 3 after transplantation.

**Fig. 3:**
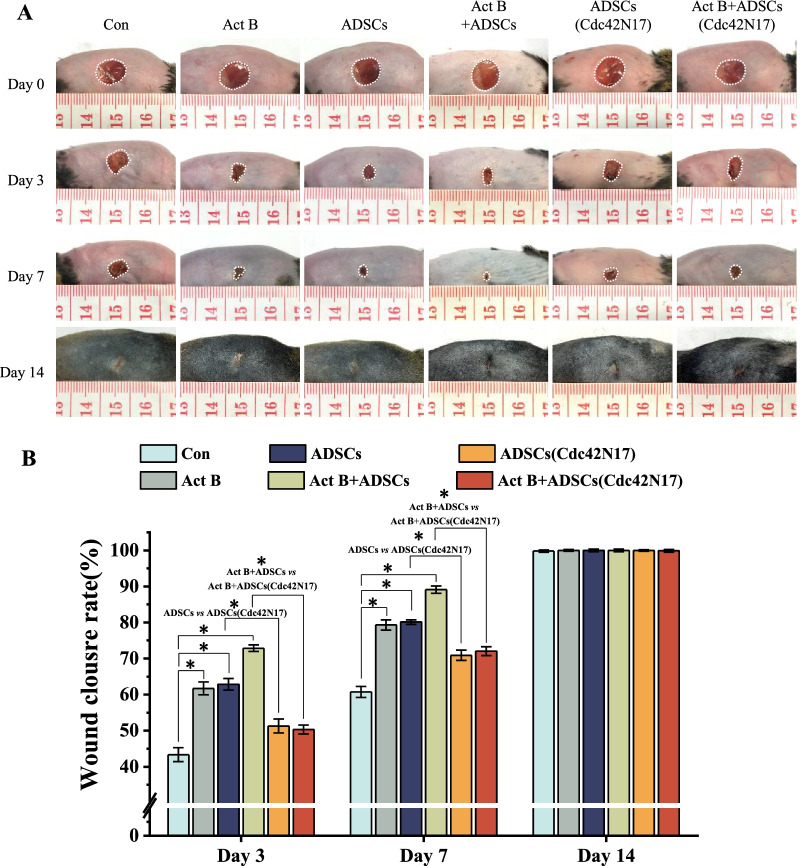
Cdc42-regulated activin B-induced ADSCs-mediated skin wound healing in vivo*.* **A** Representative macroscopic images of wounds treated with PBS, activin B, ADSCs, activin B+ADSCs, ADSCs (Cdc42N17), or activin B+ADSCs (Cdc42N17) on days 0, 3, 7, and 14. **B** Quantitative analysis of wound closure rate of six mice per group. All values are expressed as mean ± SD from six independent repeats. *P < 0.05

Wound closure rates were then evaluated. Consistently with the reported study [13], the wound closure rates in the activin B+ADSCs group were significantly accelerated compared with that of the control, activin B, or ADSCs group (Fig. 3A, B). However, the wound closure rate of ADSCs (Cdc42N17) was significantly decreased compared with that of the ADSCs group on days 3 and 7 after treatment (Fig. 3A, B). In addition, the wound healing rate in the activin B+ADSCs (Cdc42N17) group was also decreased compared with that of the activin B+ADSCs group (Fig. 3A, B). As there was no difference in wound healing rate between ADSCs (Cdc42N17) group and activin B+ADSCs (Cdc42N17) group, these data suggest that Cdc42N17 inhibited wound healing rate induced by activin B and ADSCs in vivo.

### Cdc42 promotes activin B-induced re-epithelialization, granulation tissue formation, and collagen deposition in skin wound healing

**Fig. 4:**
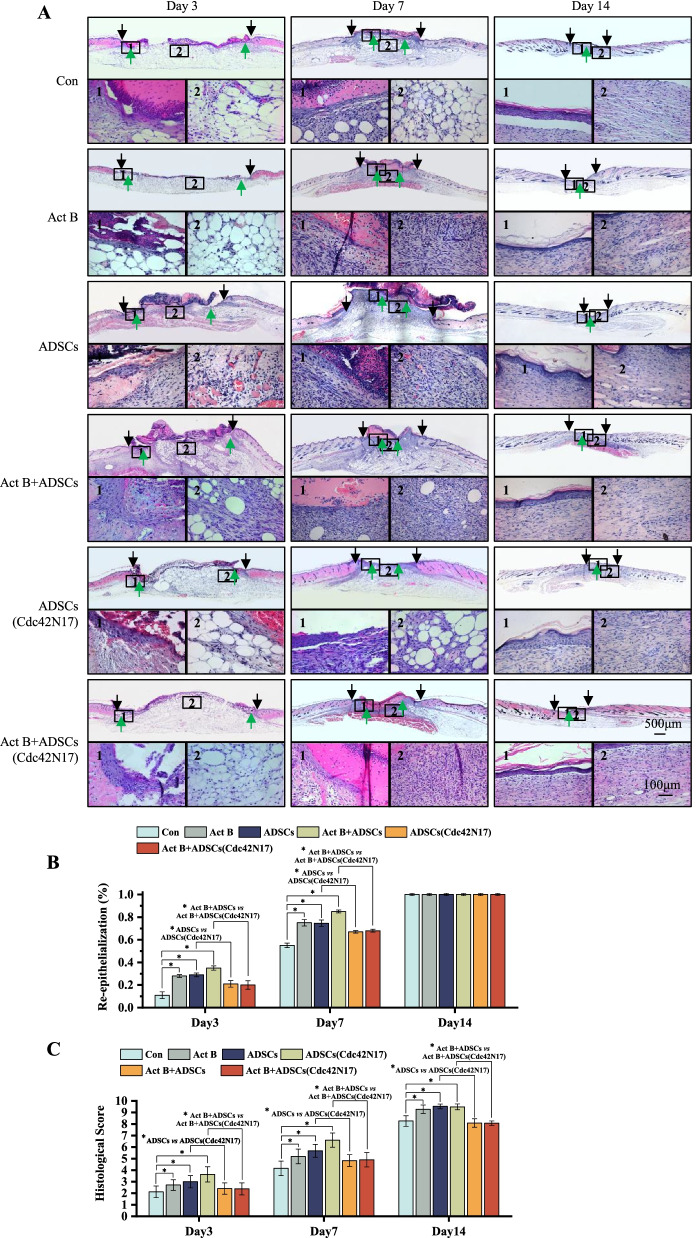
Cdc42-regulated activin B-induced ADSCs-mediated re-epithelialization and granulation tissue formation in skin wound healing. **A** H&E staining showed the re-epithelialization and granulation tissue formation of the six groups after wounding for 3, 7, and 14 days. Black and green arrows represent the dermal border and epidermal margin (scale bar = 500 μm), whereas insets of main figures (1) and (2) represent wound re-epithelialization and granulation tissue (scale bar = 100 μm), respectively. **B** The percentage of re-epithelialization ratio of six experiments. **C** Histological scores of granulation tissue thickness. All values are expressed as mean ± SD from three independent repeats. *P < 0.05

The results of H&E staining revealed that re-epithelialization in ADSCs (Cdc42N17) group was impaired compared with that of ADSCs groups on day 3 and 7 with or without activin B (Fig. 4A, B). Moreover, granulation tissue scores of ADSCs (Cdc42N17) and activin B+ADSCs (Cdc42N17) groups were much lower than those of the ADSCs and activin B+ADSCs groups, respectively. Re-epithelialization and granulation tissue scores in ADSCs (Cdc42N17) group were similar to that in activin B+ADSCs (Cdc42N17) group (Fig. 4A–C).

The Masson trichrome staining experiment was used to investigate the collagen deposition on day 7 and day 14 after treatment. After 7 days of treatment, there was little collagen deposition in the control group. In contrast, more newly formed collagen appeared in the ADSCs and activin B+ADSCs groups. However, there was still a large area of tissue without collagen deposition in the ADSCs (Cdc42N17) and activin B+ADSCs (Cdc42N17) groups. After 14 days of treatment, the collagen deposition pattern in the activin B+ADSCs group was similar to that in normal skin at the wound gap. However, the collagen deposition pattern in the ADSCs (Cdc42N17) group was similar to that in activin B+ADSCs (Cdc42N17) group, which was significantly less mature than that in the ADSCs group (Additional file 1: Fig. S4).

These data reveal that Cdc42 regulates activin B-induced re-epithelialization, granulation tissue formation, and collagen deposition or maturation after wounding.

### Cdc42 regulates the neovascularization and wound contraction in activin B-induced ADSCs-mediated skin wound healing

**Fig. 5:**
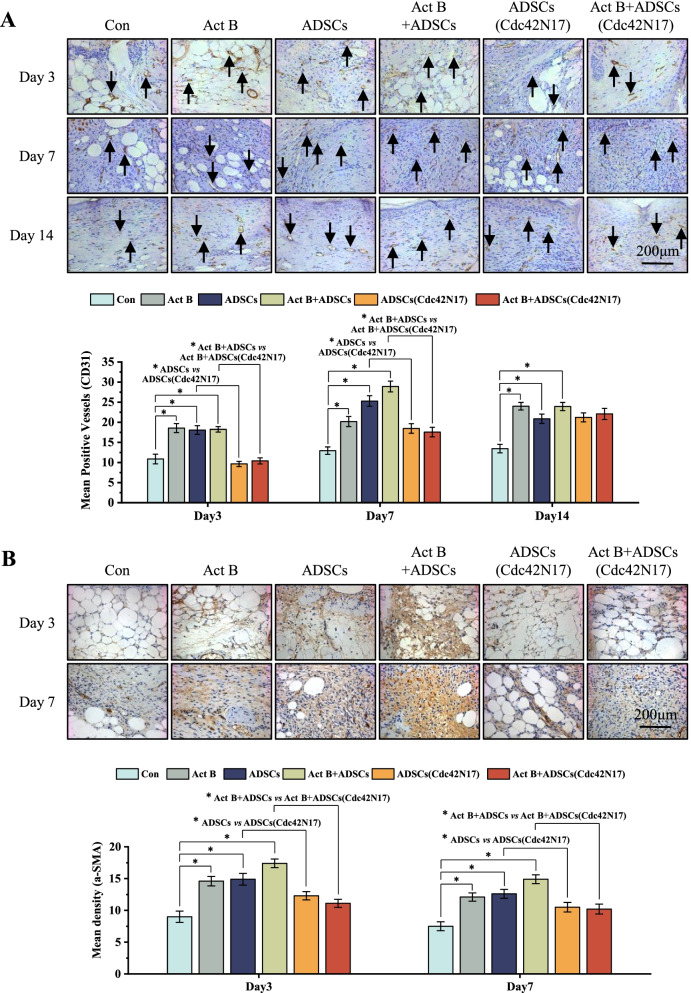
Cdc42 regulates activin B-induced ADSCs-mediated neovascularization and wound contraction in vivo. **A** Representative photomicrographs of CD 31 immunohistochemical staining treated with PBS, activin B, ADSCs, activin B+ADSCs, ADSCs (Cdc42N17), and activin B+ADSCs (Cdc42N17), respectively, at the specified time. The black arrow indicates positive staining of CD31. Scale bars = 200 μm. **B** Representative photomicrographs of α-SMA immunohistochemical staining of wounds per group on days 3 and 7 after wounding. Scale bars = 200 μm. The areas stained with α-SMA were determined by planimetric image analysis using Image-Pro Plus 6.0 software. All values are expressed as mean ± SD from three independent repeats, *P < 0.05

Immunohistochemistry staining of CD31 and α-SMA was used to evaluate the neovascularization and wound contraction. We found that the expression of CD31 and α-SMA in the wounds treated with ADSCs (Cdc42N17) was decreased compared with that of ADSCs group on days 7 and 14 (Fig. 5A, B). Similarly, less expression of CD31 and α-SMA in activin B+ADSCs (Cdc42N17) group was observed compared with that of activin B+ADSCs group (Fig. 5A, B). Cdc42N17 inhibited CD31 and α-SMA expression in activin B+ADSCs group to the levels comparable to that in ADSCs (Cdc42N17) group (Fig. 5A, B). These data suggest that Cdc42 promotes the neovascularization and wound contraction in activin B-induced ADSCs-mediated skin wound healing.

### RNA sequencing identifies possible mechanism of Cdc42 in the regulation of ADSCs biological function

**Fig. 6:**
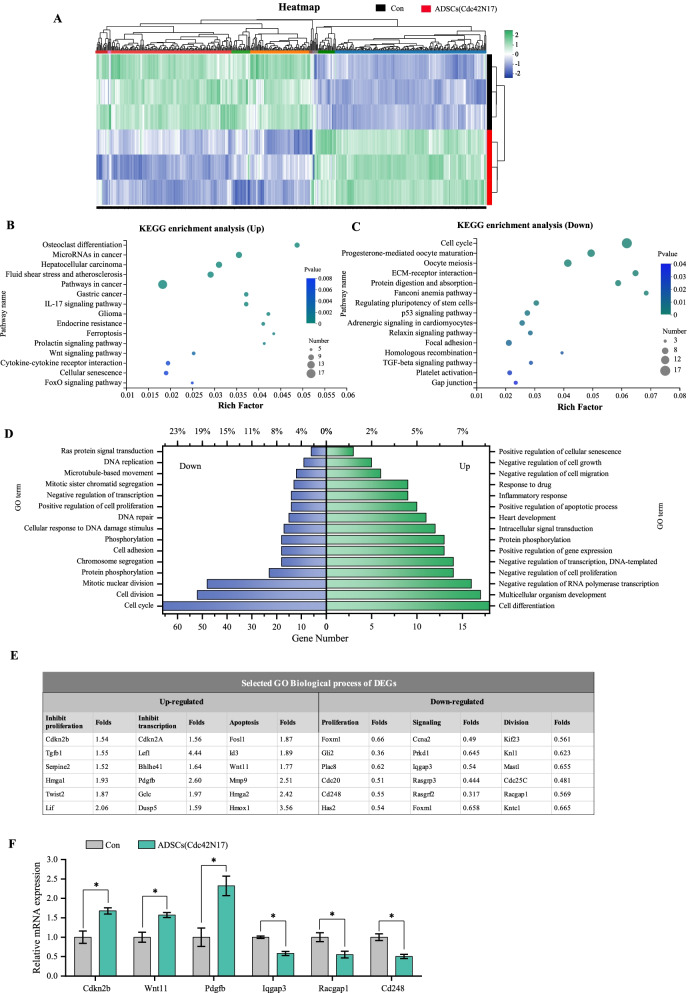
Cdc42 deletion in ADSCs exhibits a distinct transcriptional signature. **A** Heatmap showing differentially expressed genes (DEGs) between ADSCs (Con) and ADSCs transduced with Cdc42N17. There were 216 upregulated and 266 downregulated DEGs in ADSCs (Cdc42N17), respectively, and were subjected to Kyoto Encyclopedia of Genes and Genomes pathways enriched (KEGG) and gene ontology (GO) analysis. **B**, **C** showing the top 15 enrich pathways in upregulated and downregulated DEGs, while **D** showing the top 15 biological processes in upregulated and downregulated DEGs. **E** Selected gene expression in some of the GO biological processes. **F** qPCR analysis of relative expression level of *Cdkn2b*, *Wnt11*, *Pdgfb*, *Iqgap3*, *Racgap1* and *Cd248* in ADSCs (Cdc42N17). All values are expressed as mean ± SD from three independent repeats. *P < 0.05

RNA sequencing was further conducted in ADSCs group and ADSCs (Cdc42N17) group to explore global gene expression changes induced by Cdc42N17. By principal component analysis (PCA), we found that there was a strong correlation between biological duplicate samples (Additional file 1: Fig. S5). Compared with ADSCs group, a total of 216 upregulated and 266 downregulated DEGs were detected in ADSCs (Cdc42N17) (Fig. 6A).

The KEGG pathway enrichment analysis found that compared with ADSCs group, the upregulated DEGs in ADSCs (Cdc42N17) group were enriched in microRNAs in cancer, pathways in cancer, wnt, cytokine–cytokine receptor interaction, cellular senescence, and foxo (Fig. 6B). The downregulated DEGs in ADSCs (Cdc42N17) were enriched in the pathways of cell cycle, p53, regulating pluripotency of stem cells, focal adhesion, TGF-β, and gap junction pathways (Fig. 6C).

The GO enrichment analysis revealed that compared with ADSCs group, the upregulated DEGs in ADSCs (Cdc42N17) group were significantly associated with cell differentiation, negative regulation of cell proliferation, transcription and cell migration, positive regulation of apoptotic process, and intracellular signal transduction (Fig. 6D). On the other hand, the downregulated DEGs were associated with cell cycle, cell division, mitotic nuclear division, cell adhesion, and cell proliferation (Fig. 6D).

We listed the upregulated and downregulated genes in some of the GO enrichment terms (Fig. 6E). The expression levels of *Cdkn2b, Wnt11, Pdgfb, Iqgap3, Racgap1*, and *Cd248* were verified by qPCR, and the results were consistent with the RNA-seq (Fig. 6F).

Taken together, these data suggest that Cdc42 plays a role in cell proliferation, migration, adhesion, and cell cycle of ADSCs. Furthermore, the signaling in wnt, foxo, cell cycle, p53, and TGF-β pathway may contribute to Cdc42-regulated biological function of ADSCs.

### RNA sequencing identifies the possible mechanism of Cdc42 in the regulation of activin B-induced ADSCs biological function

**Fig. 7:**
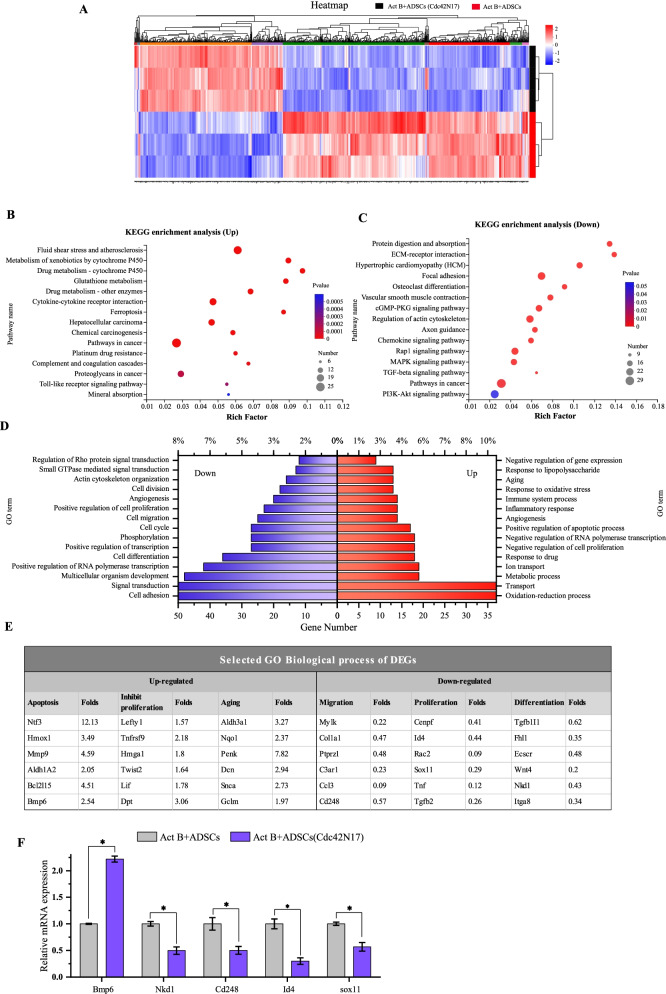
Cdc42 regulates activin B-induced ADSCs transcriptional signature. **A** Heatmap showing differentially expressed genes (DEGs) between activin B+ADSCs and activin B+ADSCs (Cdc42N17). There were 355 upregulated (Red) and 615 (Blue) downregulated DEGs in activin B+ADSCs (Cdc42N17), respectively, and were subjected to Kyoto Encyclopedia of Genes and Genomes pathways (KEGG) enriched and gene ontology (GO) functional enrichment analysis. **B** and **C** showing the genes top 15 enrich pathway, while **D** showing the top 15 GO terms in upregulated and downregulated DEGs. **E** Selected gene expression in some of the GO terms. **F** qPCR analysis of relative expression level of *Bmp6*, *Nkd1*, *Cd248*, *Id4*, and *Sox11* in Act B+ADSCs (Cdc42N17). All values are expressed as mean ± SD from three independent repeats. *P < 0.05

To further identify the mechanism of Cdc42 in the regulation of activin B-induced ADSCs biological function, RNA sequencing was performed in activin B+ADSCs group and activin B+ADSCs (Cdc42N17) group. There was obvious difference between the two groups (Additional file 1: Fig. S6). Activin B+ADSCs (Cdc42N17) group showed 355 upregulated DEGs and 615 downregulated DEGs (Fig. 7A).

KEGG pathway enrichment analysis revealed that the upregulated DEGs were enriched in the pathway of metabolism of xenobiotics by cytochrome P450, drug metabolism—cytochrome P450, glutathione metabolism, cytokine–cytokine receptor interaction, and proteoglycans in cancer (Fig. 7B). The downregulated DEGs were enriched in the pathway of ECM–receptor interaction, focal adhesion, Rap1, MAPK, TGF-β, and PI3K-Akt (Fig. 7C).

In the GO annotations analysis, the upregulated DEGs were enriched in the biological processes of oxidation–reduction process, transport, negative regulation of cell proliferation, and aging (Fig. 7D). The downregulated DEGs were enriched in the biological processes of cell adhesion, signal transduction, positive regulation of cell proliferation and migration, cell cycle, and angiogenesis (Fig. 7D). The detailed selected gene expression changes in the biological processes of apoptosis, negative regulation of proliferation, aging, migration, proliferation, and differentiation are shown in Fig. 7E.

Moreover, the changes in selected gene expression such as *Bmp6*, *Nkd1*, *Cd248*, *Id4*, and *Sox11* were confirmed by qPCR (Fig. 7F).

Taking together, these data imply that Cdc42 plays a role in activin B-induced signal transduction, cell adhesion, cell migration, and cell proliferation of ADSCs. Furthermore, the signaling pathway of PI3K-Akt, pathway in cancer, focal adhesion, Rap1, MAPK, and TGF-β may contribute to Cdc42’s regulation of activin B-induced biological function of ADSCs.

### The Erk-Srf pathway is involved in Cdc42-mediated activin B-induced ADSCs proliferation

**Fig. 8:**
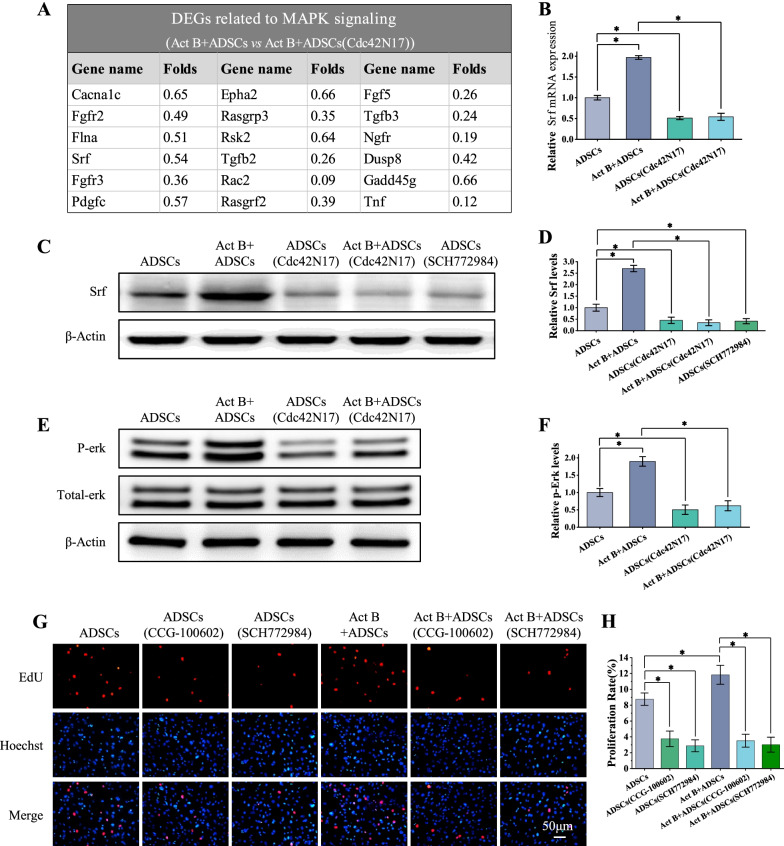
Activin B-induced ADSCs proliferation by activating Cdc42-Erk-Srf pathway. **A** Genes expression related to MAPK signaling in Act B+ADSCs (Cdc42N17) group. **B** qPCR analysis was performed to confirm the relative mRNA expression level of *Srf* in four groups. **C**, **E** Western blot assays were performed to detect the level of Srf and phosphorylation Erk (p-Erk) in ADSCs, activin B+ADSCs, ADSCs (SCH772984) (Erk1/2 inhibitor) or ADSCs (Cdc42N17) treated with or without activin B, respectively. **D**, **F** The band and the relative quantification levels of the expression of Srf and phosphorylation Erk were analyzed. **G** Representative fluorescence imaging of EdU staining of ADSCs and ADSCs treated with Srf inhibitor CCG-100602 or Erk1/2 inhibitor SCH772984 and with/without activin B at 6 h. Scale bar = 50 μm. **H** The proliferation rates were quantified by calculating the percentage of EdU-positive cells. All values are expressed as mean ± SD from three independent repeats. *P < 0.05

Among the differentially expressed genes in the activin B+ADSCs and activin B+ADSCs (Cdc42N17) group, several members of the MAPK signaling pathways were downregulated in activin B+ADSCs (Cdc42N17) group (Fig. 8A). We confirmed the downregulation of Srf in activin B+ADSCs (Cdc42N17) group by qPCR and western blot (Fig. 8B, C). For Srf is a major downstream cytosolic transcription factor of Erk/MAPK signaling [41], the effect of Cdc42 on Erk/MAPK activity and Srf expression was further explored. The result of qPCR and western blot confirmed that while Erk and its downstream Srf were increased upon activin B treatment, this increase was abolished by inhibition of Cdc42 (Fig. 8B–F). Further study of the mechanism revealed that the activation of Erk signal in ADSCs by activin B was able to induce proliferation. However, Erk inhibitor SCH772984 abolished the growth of ADSCs with activin B (Fig. 8G, H). The inhibition of Srf by CCG-100602 also suppressed ADSCs proliferation induced by activin B (Fig. 8G, H). These data suggest that Erk-Srf pathway is involved in Cdc42-mediated activin B-induced ADSCs proliferation.

## Discussion

We have previously found that activin B is able to induce migration of ADSCs to promote skin wound healing [13]. However, whether Cdc42 signaling is important in ADSCs remained unknown. The current study found that Cdc42 signaling is essential for ADSCs-mediated cutaneous wound healing as well as proliferation and secretion*.* While our previous study found that Cdc42 regulates BMSCs-mediated wound healing by affecting Golgi reorientation [20], this study found that Cdc42 regulates ADSCs-mediated cutaneous wound healing by affecting VEGF-mediated vascularization, myofibroblast differentiation-mediated granulation tissue formation, and Erk-Srf signaling pathway.

Cutaneous wound healing is a dynamic process that involves overlapping phases, including inflammation, granulation tissue formation, and matrix remodeling [42]. ADSCs have been shown to enhance wound healing via their paracrine function [43]. The present study suggests that Cdc42 signaling is partially responsible for activin B-induced secretion of VEGF in ADSCs. VEGF stimulates wound healing through angiogenesis [44]. Consistently, we have found that the ADSCs-mediated vascularization in cutaneous wound healing is suppressed upon Cdc42 suppression, as the expression of CD31 is decreased in ADSCs (Cdc42N17) group induced by activin B. These data suggest that Cdc42 regulates activin B-induced ADSCs-mediated vascularization in cutaneous wound healing via regulation of VEGF secretion.

Myofibroblast, mostly characterized by high expression of α-smooth muscle actin (α-SMA), is the major cell type responsible for ECM production during wound healing [45]. A recent study has shown that activin A promotes myofibroblast differentiation of endometrial mesenchymal stem cells [46]. In the present study, we found that activin B may also promote myofibroblast differentiation of ADSCs, as evidenced by an up-regulation of the expression of α-SMA in the wounds of activin B+ADSCs group. In addition, we have found that the expressions of α-SMA in the wounds treated with ADSCs (Cdc42N17) were decreased compared with that of ADSCs group. Furthermore, our animal studies have revealed that the wound granulation tissue formation is impaired upon Cdc42 suppression. These data indicate that Cdc42 regulates the ADSCs-mediated granulation tissue formation in cutaneous wound healing via regulation of myofibroblast differentiation. In line with this, Ge’s study shows that Cdc42 is required for TGFβ1-induced α-SMA expression in MSCs [18]. They have found that TGFβ1-induced expression of α-SMA is significantly decreased in Cdc42 knockout BMSCs after 24-h stimulation [18]. Hence, Cdc42 is necessary for myofibroblast differentiation of MSCs (e.g., BMSCs and ADSCs).

Comprehensive understanding of the mechanism of ADSCs in the wound healing process is of great significance for further improving wound healing effect. Our RNA sequencing begins to reveal potential mechanism of Cdc42 in the regulation of activin B-induced ADSCs biological function. The RNA sequencing results provide evidence that Cdc42 globally controls gene expression pattern of ADSCs treated with/without activin B and indicate possible signaling pathways downstream of Cdc42 that is associated with the regenerative ability of ADSCs. The Erk/MAPK pathway is an essential intracellular signal transduction pathway that controls cell proliferation [47, 48]. The previous study showed that exendin-4 treatment promotes ADSCs growth via ERK signaling pathways [49]. In the present study, we found that activin B contributes to ADSCs proliferation via Cdc42-Erk-Srf pathway. Our findings may help understand the comprehensive molecular mechanisms of ADSCs in wound healing.

## Conclusions

The present study shows that Cdc42 is a key regulator of ADSCs-mediated cutaneous wound healing induced by activin B. Firstly, Cdc42 regulates activin B-induced migration and proliferation in ADSCs. Secondly, Cdc42 regulates ADSCs-mediated vascularization in cutaneous wound healing via regulation of VEGF secretion. Thirdly, Cdc42 regulates ADSCs-mediated granulation tissue formation in cutaneous wound healing via regulation of myofibroblast differentiation. In addition, RNA sequencing further identifies the potential mechanism of Cdc42 in the regulation of activin B-induced ADSCs biological function. Last but not the least, activin B might activate the Cdc42-Erk-Srf signaling pathway to promote the proliferation of ADSCs in the skin wound healing process. This study significantly advances the understanding of the function of Cdc42 in the ADSCs-mediated cutaneous wound healing.

## Supplementary Information

**Additional file 1**. Experimental details (characterization of ADSCs; the detail methods for dehydration, paraffin embedding, and H&E; GST pull-down assay and western blot); the criteria for histological evaluation of cutaneous wound healing; the primers used in this study; characterization of ADSCs; ADSCs transduced with lentivirus containing Cdc42N17, Cdc42L61, and the EGFP vector; ADSCs participated in cutaneous wound healing; Cdc42 regulates activin B-induced collagen deposition and maturation after wounding; gene expression patterns analysis between ADSCs and ADSCs (Cdc42N17); gene expression patterns analysis between activin B+ADSCs and activin B+ADSCs (Cdc42N17).

s

## Data Availability

The raw sequence data reported in this paper have been deposited in the Genome Sequence Archive (Genomics, Proteomics & Bioinformatics 2021) in National Genomics Data Center (Nucleic Acids Res 2022), China National Center for Bioinformation/Beijing Institute of Genomics, and Chinese Academy of Sciences (GSA: CRA006666) that are publicly accessible at https://ngdc.cncb.ac.cn/gsa. All data generated or analyzed during this study are included in this published article and its Additional file 1.

## Abbreviations

ADSCs: Adipose-derived mesenchymal stem cells
Act B: Activin B
Cdc42N17: Cdc42 dominant-negative mutant
Cdc42L61: Cdc42 constitutively active mutant
Srf: Serum response factor
Erk: Extracellular signal-regulated kinase
MAPK: Mitogen-activated protein kinase
H-DMEM: High-glucose Dulbecco’s modified Eagle’s medium
PBS: Phosphate-buffered saline
DAPI: 4,6-Diamidino-2-phenylindole
EdU: 5-Ethynyl-2’-deoxyuridine
IHC: Immunohistochemistry
SDS-PAGE: Sodium dodecyl sulfate–polyacrylamide gel electrophoresis
RNA-seq: RNA sequencing
DEGs: Differential expression genes
GO: Gene ontology
KEGG: Kyoto Encyclopedia of Genes and Genomes
qPCR: Quantitative real-time polymerase chain reaction
H&E: Hematoxylin and eosin
