# Supplementary material for: Activin B-activated Cdc42 signaling plays a key role in regulating adipose-derived mesenchymal stem cells-mediated skin wound healing
Source: Stem Cell Res Ther. 2022 Jun 11;13:248. doi: 10.1186/s13287-022-02918-9 (PMC9188063; doi:10.1186/s13287-022-02918-9)
Supplement: Supplementary file 1 — Additional file 1. Experimental details (characterization of ADSCs; the detail methods for dehydration, paraffin embedding, and H&E; GST pull-down assay and western blot); the criteria for histological evaluation of cutaneous wound healing; the primers used in this study; characterization of ADSCs; ADSCs transduced with lentivirus containing Cdc42N17, Cdc42L61, and the EGFP vector; ADSCs participated in cutaneous wound healing; Cdc42 regulates activin B-induced collagen deposition and maturation after wounding; gene expression patterns analysis between ADSCs and ADSCs (Cdc42N17); gene expression patterns analysis between activin B+ADSCs and activin B+ADSCs (Cdc42N17). [file 13287_2022_2918_MOESM1_ESM.pdf]

Activin B-activated Cdc42 signaling plays a key role in regulating adipose-derived mesenchymal stem cells-mediated skin wound healing

Simin Huang[a,1], Xueer Wang[a,1], Min Zhang[a], Mianbo Huang[a], Yuan Yan[a], Yinghua Chen[a], Yijia Zhang[a], Jinfu Xu[a], Lingwei Bu[a], Ruyi Fan[a], Huiyi Tang[a], Canjun Zeng[c], Lu Zhang[b, *], Lin Zhang[a, **]

[a,] Department of Histology and Embryology, NMPA Key Laboratory for Safety Evaluation of Cosmetics, Key Laboratory of Construction and Detection in Tissue Engineering of Guangdong Province, School of Basic Medical Sciences, Southern Medical University, Guangzhou, 510515, People's Republic of China.

[b,] Key Laboratory of Functional Proteomics of Guangdong Province, Key Laboratory of Mental Health of the Ministry of Education, School of Basic Medical Sciences, Southern Medical University, Guangzhou 510515, China.

[c,] Department of Orthopedics, Third Affiliated Hospital of Southern Medical University, Academy of Orthopedics Guangdong Province, Guangzhou, Guangdong, 510630, China.

[*,] Corresponding author. E-mail address: zlulu70@126.com.

[**,] Corresponding author. E-mail address: zlilyzh@126.com

[1,] These authors are contributed equally to this work.

Supporting information

1. Characterization of adipose-derived mesenchymal stem cells.

Immunofluorescence staining.

Cells of ADSCs at passage 3 were harvested with 0.25% trypsin, then, seeded on cover glass in 24-well plates (Corning) at a density of 5 × 10^5 cells per well. When the cells reach approximately 90% confluent, the medium was discarded and washed twice with D-PBS before and after fixed with 4% paraformaldehyde solution (Cat# G0528, Gbcbio, China) for 20 min at room temperature, respectively. After permeabilized with 0.5% Triton X-100 solution for 15 min, they were blocked in 5% BSA for 10 min and incubated in phallacidin for 30 min. Following incubating with a primary antibody against CD44 (1:500, Cat# ab157107, Abcam), CD90 (1:1000, Cat# ab3105, Abcam),

CD31 (1:50, Cat# ab281583, Abcam) and CD80 (1:100, Cat# ab254579, Abcam) at 4 °C for overnight, cells were rinsed twice with PBS. Then incubated with a fluorescently labeled secondary antibody: goat anti-rabbit IgG (1:500, Cat#A-11008, Invitrogen) and goat anti-Rat IgG (1:500, Cat#A-11006, Invitrogen) at room temperature for 1 h, washed twice and stained with Hoechst 33258 (1:500, Cat# H21491, Invitrogen) for 15 min before inverted fluorescence microscope investigate

Differentiation induction experimentation.

(1) Osteogenic induction differentiation

To verfied the cells multipul differentiation ability, mouse adipose-derived mesenchymal stem cell osteogenic differentiation kit (Cat# MUXMD-90021, Cyagen, China) were use to detect osteogenic induction differentiation ability. To be specific, cells of ADSCs at passage 3 were harvested with 0.25% trypsin, then, seeded in a Gelatin-coated 24-well plate at a density of 5×10^5 cells per well. When the cells reach approximately 70% confluent, the medium was discarded and washed twice with D-PBS and replace with osteogenic differentiation complete medium. The culture medium was updated every 72 h. After culture for 4 weeks, the medium was discarded and washed twice with D-PBS and later fixed with 4% paraformaldehyde solution for 20 min at room temperature. Alizarin Red stain solution was used to stain the calcium nodules and samples were observed by the microscope.

(2) Adipogenic induction differentiation

To furether verfied the cells multipul differentiation ability, mouse adipose-derived mesenchymal stem cell adipogenic differentiation kit (Cat# MUXMD-90031, Cyagen, China) were use to detect adipogenic induction differentiation ability. To be specific, cells of ADSCs at passage 3 were harvested with 0.25% trypsin, then, seeded in a Gelatin-coated 24-well plate at a density of 5×10^5 cells per well. When the cells reach approximately 100% confluent, the medium was discarded and washed twice with D-PBS and replace with adipogenic induction differentiation medium A. After culture for 3 days, the medium was replaced by adipogenic induction differentiation medium B and culture for another 24 h. After five circles by medium A and B, cells were maintaining by medium B for another 7 days and update every 24 h. Oil red O stain solution was used to detect the adipogenic differentiation effect and image was capture by the microscope.

(3) Chondrogenic induction differentiation.

To furether verfied the cells multipul differentiation ability, mouse adipose-derived mesenchymal stem cell chondrogenic differentiation kit (Cat# MUXMD-90041, Cyagen, China) were use to detect chondrogenic induction differentiation ability. To be specific, cells of ADSCs at passage 3 were harvested with 0.25% trypsin, then, 4 × 10^5 cells were transfer to a 15 ml centrifuge tube, the medium was discarded and washed twice with chondrogenic differentiation premix, and culture with chondrogenic differentiation complete medium. The culture medium was updated every 72 h. After culture for 4 weeks, when the cells form cartilage balls with a diameter of about 2 mm, the medium was discarded and washed twice with D-PBS and later fixed with 4% paraformaldehyde solution for 30 min at room temperature. The sample was dehydrated in a graded increase ethanol series (50%, 70%, 80%, 95% and 100% each for 0.5 h), then cleared in dimethylbenzene and ethanol mixture (1:1) for 2.0 h, follow by dimethylbenzene twice for 1.5 h. Before embedded in paraffin, these samples were steeped in the paraffin and dimethylbenzene mixture for 1.0 h at 40 °C, then in pure paraffin at 55 °C for 0.5 h. Then these paraffin-embedded samples were sliced into 3 μm, adhered to glass slide, and placed in an oven at 35°C overnight. Selected several slices for alcian blue staining, after deparaffinized in dimethylbenzene twice for 10 min and rehydrated in the following graded decreased ethanol series for 10 min (100%, 100%, 95%, 85%, 70% and 50%). Next, the samples were staining by alcian blue 8GX solultion for 1.0 h at 37 °C. Finally, after wash by water for 5 min, the samples were observed by the microscope (Leica DM 4000B).

Flow Cytometry assay

Cells of ADSCs at passage 3 were harvested with 0.25% trypsin, then, washed twice with D-PBS and re-suspended with flow cytometry buffer. Adjust the cell concentration to 3 × 10^6 cells/ml and 100 μl of cell suspension transfer into each flow cytometry tube (about 3×10^5 cells). Following incubating with a primary antibody against CD29 (Cat# 102202, Biolegend), CD31 (Cat# 102402, Biolegend), CD44 (Cat# 103002, Biolegend), CD45 (Cat# 103104, Biolegend), CD90.2 (Cat# 140302, Biolegend) and CD117 (Cat# 105802, Biolegend) at 4°C for 30 min. Then the medium was discarded and cells were rinsed twice with flow cytometry buffe and re-suspended in 100 μl flow cytometry buffer. Then incubated with an immunoglobulin fluorochrome conjugated secondary antibody: goat anti-Rat IgG (1:50, Cat# 405404, Biolegend) and goat anti- hamster IgG (1:50, Cat# 405502, Biolegend) at 4°C for 30 min in the dark. Next, washed twice with flow cytometry buffe and resuspending the cells with 500 μl

PBS. At last, single-cell suspension was prepared and then the cell surface markers on ADSCs were analyzed by LSRFortessa™ X-20 flow cytometry (BD Pharmingen). The flow cytometry data were analyzed using ModFit LT 5.0 software (Verity Software House, Topsham).

CFSE labeling

For 5, 6-carboxyfluorescein diacetate succinimidyl ester (CFSE) labeling, ADSCs at passages 3 were serum-free starve for 12 h and 5 μM CFSE (diluted in 0.1 M PBS) (Cat# C1157, Invitrogen) was added and allowed to incubate at 37°C for 30 min. Following CFSE removal, fresh culture medium was added, and the cells were further incubated at 37°C for 30 min.

2. The detail methods for dehydration, paraffin embedding and H&E

After dividing the wound in half along the longest diameters margin, the sample was dehydrated in a graded increase ethanol series (50% for 2 h, 70% overnight, 80%, 90% and 95% each for 1 h, 100% for 0.5 h and repeat 100% for 0.5 h), then cleared in dimethylbenzene twice for 2-3 min. Before embedded in paraffin, these samples were steeped in the following paraffin for 1 h: 48~50 °C, then 54-56 °C, 58-60 °C. Then these paraffin-embedded samples were sliced into 5 μm, adhered to glass slide, and placed in an oven at 40°C overnight.

For H&E staining, selected several slices adjacent to the wound center for histological assessment after deparaffinized in dimethylbenzene twice for 10 min and rehydrated in the following graded decreased ethanol series for 5 min (100%, 100%, 90%, 80% and 70%). Firstly, the samples were steeped in hematoxylin and wash with water for 10 min each, then soaked for 5 min with 70% and 80% alcohols in sequence followed by staining with eosin for 1 min. After dehydrated in a graded ethanol series (90%, 95% and 95% for 5 min, 100% and 100% for 10 min) and cleared in dimethylbenzene twice for 10 min, they were sealed with neutral gum. Finally, the samples were observed by the microscope (Leica DM 4000B).

3. GST pull-down assay and western blot analysis

Cells were washed twice with ice-cold tris-buffered saline (TBS) and lysed with ice-cold Mg^{2+} containing buffer (MLB: 25 mM HEPES, pH 7.5, 150 mM NaCl, 1% Igepal CA-630, 10% glycerol, 25 mM NaF, 10 mM $MgCl_2$, 1 mM EDTA, 1 mM sodium orthovanadate and 1 mM PMSF). The cells were rapidly scraped off by a cell scraper and collected to a precooled tube. Total protein concentrations were determined by Enhanced BCA Protein Assay Kit (Cat# P0010, Beyotime, China). The cell lysates were

divided into two equal parts, one was blotted for total Cdc42 using anti-Cdc42 antibody (1:2000, Cat# ab187643, Abcam) and HRP-conjugated anti-rabbit IgG secondary antibody (1:2000, Cat# ab6721, Abcam), the other separate cell lysate was subjected to a PAK1 PBD binding assay. To be specific, the cell lysates were centrifuged at 14,000×g for 5 min at 4 °C. Equal amounts of the clarified cell lysates for each group were incubated with a 10 μg/ml GST- PAK1 PBD agarose at 4 °C for 1 h with gentle agitation, followed by brief centrifugation at 14,000×g for 10 s at 4 °C to attain the sediment. The beads attached to the tube bottom were washed three times by centrifugation at 14,000×g for 10 s 4 °C, with 500 μl ice-cold MLB. The beads with Cdc42-GTP were then re-suspended in 40 μl 2 × laemmli sample buffer and boiled for 5 min. The beads were pelleted by brief centrifugation, and the Cdc42-GTP were analyzed by western blot using anti-Cdc42 antibody (1:2000, Cat# ab187643, Abcam) and HRP-conjugated anti-rabbit IgG secondary antibody (1:2000, Cat# ab6721, Abcam). The bands were visualized using the enhanced chemiluminescence (ECL) detection system and Gel Image System (Tanon-5200CE) was used to detect the band intensity.

Table S1 Criteria for histological evaluation of cutaneous wound healing

Project	Scores	Evaluation
**Granulation tissue**	1-3	Thin granular layer: 0–30/Hp* of fibroblasts, collagen fibers without direction, mainly inflammatory cells, only granulation tissue around the wound edge.
	4-6	Moderate granulation layer: 30–50/Hp of fibroblasts, collagen fibers arranged orderly, some capillaries or collagen deposition. granulation tissue cover 30%-50% of the wound bed.
	7-9	Thick granulation layer: 50–80/Hp of fibroblasts, a large amount of collagen fibers arranged orderly. Medium-thickness granulation tissue can range from mainly inflammatory cells to more fibroblasts and collagen deposition. Extensive neovascularization. Wound bed with granulation tissue >50%.
	10-12	Very thick granulation layer: more than 80 Hp of fibroblasts, excessive collagen fibers. Thick vascular granulation tissue dominated by fibroblasts and extensive collagen deposition. The epithelium partially to completely covers the wound.

*Note: Hp: 40×.

Table S2 Primers used in this study

Num	Genes	Forward Primer (5'to3')	Reverse Primer (5'to3')
1	***Bmp6***	ACGCCCTGTCCAATGACG	CTTGCGGTTCAAGGAGTGTG
2	***Nkd1***	CAGACTGCTACCACCATTGCG	GATCCCGGTCCAAACTGAGAC
3	***Cd248***	CTCTGTTATTTCAGCTACACGC	GCACATGACGGCAACTCCAGAG
4	***Sox11***	GGACAGCGAGAAGATCCCGT	GTCCGTCTTGGGCTTTTTGC
5	***Id4***	GCGATATGAACGACTGCTACAG	GATCTCCACTTTGCTGACTTTC
6	***Wnt11***	GGCAGCCTTGGCACTGAATC	ACGATGGTGCGCATGAGCT
7	***Cdkn2b***	CGCCCAATCCAGGTCATGAT	GCACAGGTCTGGTAAGGGTG
8	***Pdgfb***	GTCCAGGTGAGAAAGATTGAGA	GTCATGGGTGTGCTTAAACTTT
9	***Iqgap3***	CATCTCCTCCTTGTGGCTGCTAAG	GCTTGTGTCCTCGTTGGCTCTG
10	***Racgap1***	GAAGTCAGGACCTTTACAACCT	CCCAAATTGTCTGTGTCAGTTC
11	***Srf***	TGATTCAGACCTGCCTCAACTC	TGACTGTGAATGCTGGCTTC
12	***GAPDH***	TTGGCATTGTGGAAGGGCTC	ATCACGCCACAGCTTTCCAG

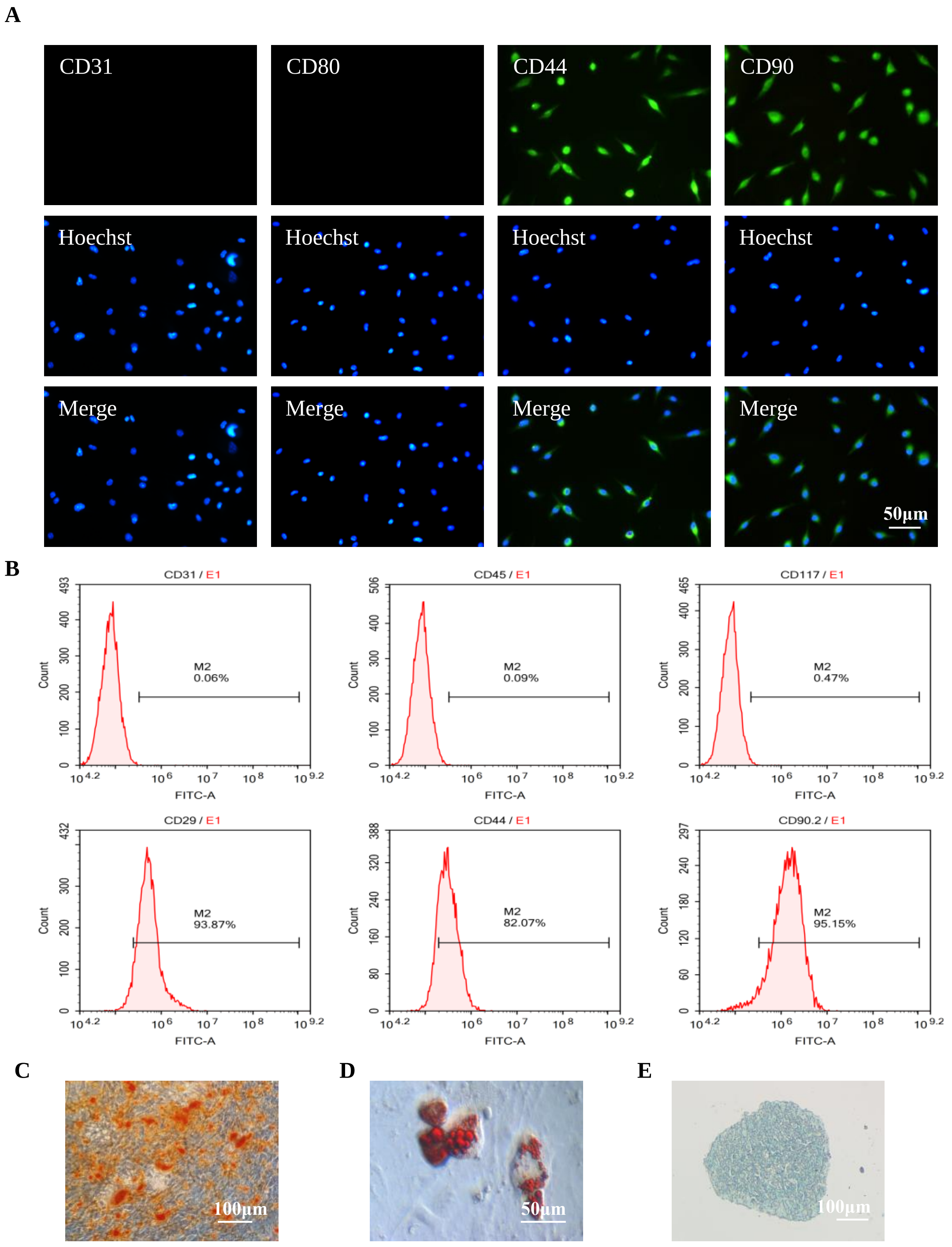

Figure.S1 Characterization of adipose-derived mesenchymal stem cells (ADSCs). A Representative fluorescence imaging of Immunocytochemistry staining for cell surface markers of CD44, CD90, CD31 and CD80. **B** Flow Cytometry was use to analyze cell surface markers of CD29, CD31, CD44, CD45, CD90.2 and CD117. Differentiation assay were performed to detect the ability of differentiation in to Osteogenic **C**, Adipogenic **D** and Chondrogenic **E**.

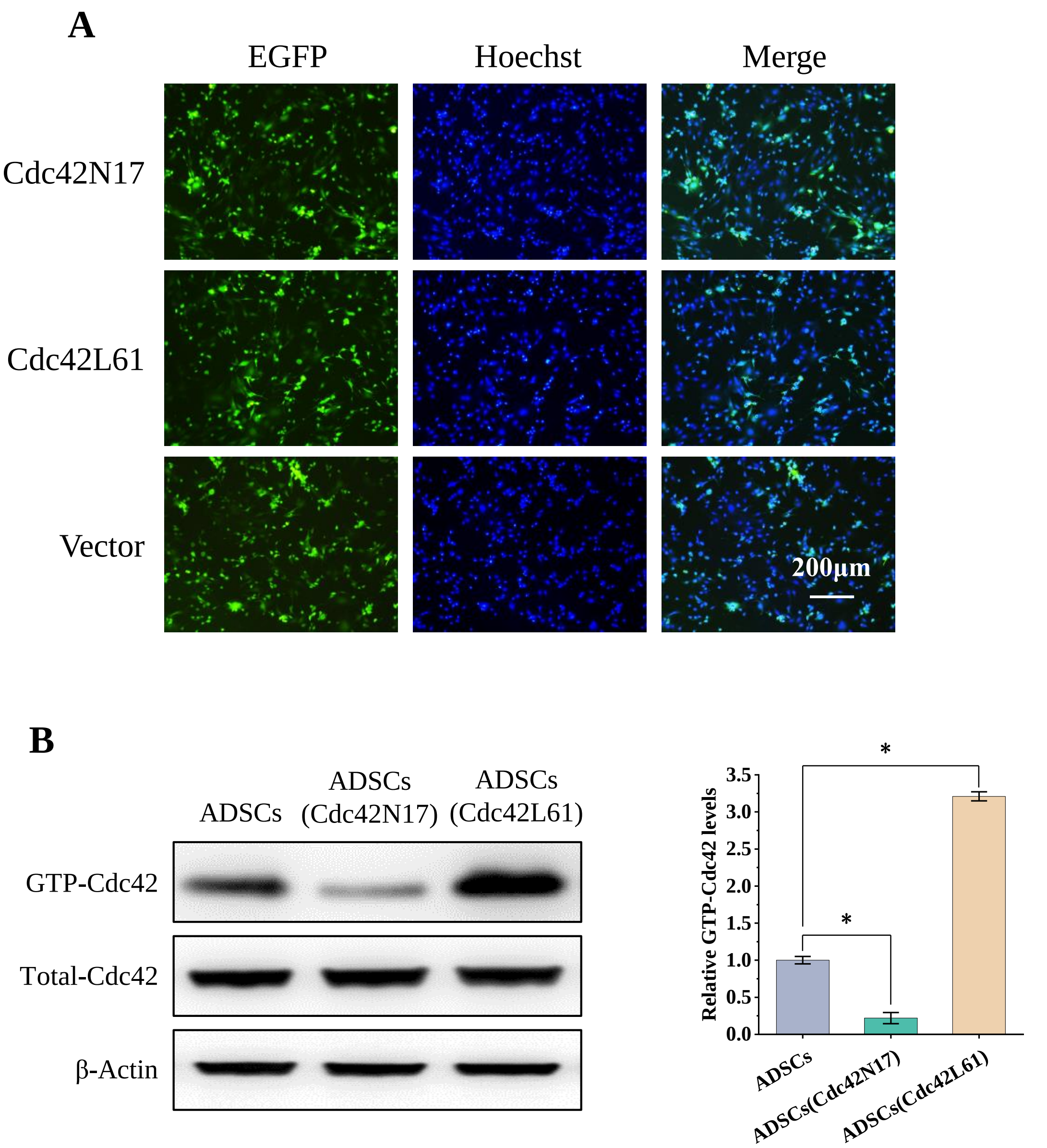

Figure.S2 ADSCs transduced with lentivirus containing the dominant negative Cdc42 mutant (Cdc42N17), the constitutively active mutant (Cdc42L61), and the EGFP lentiviral vector. A Representative fluorescence imaging of cells infected with Cdc42N17, Vector and Cdc42L61 lentivirus. **B** Pull-down assay detects changes of Cdc42 activity in ADSCs, ADSCs (Cdc42N17) and ADSCs (Cdc42L61).

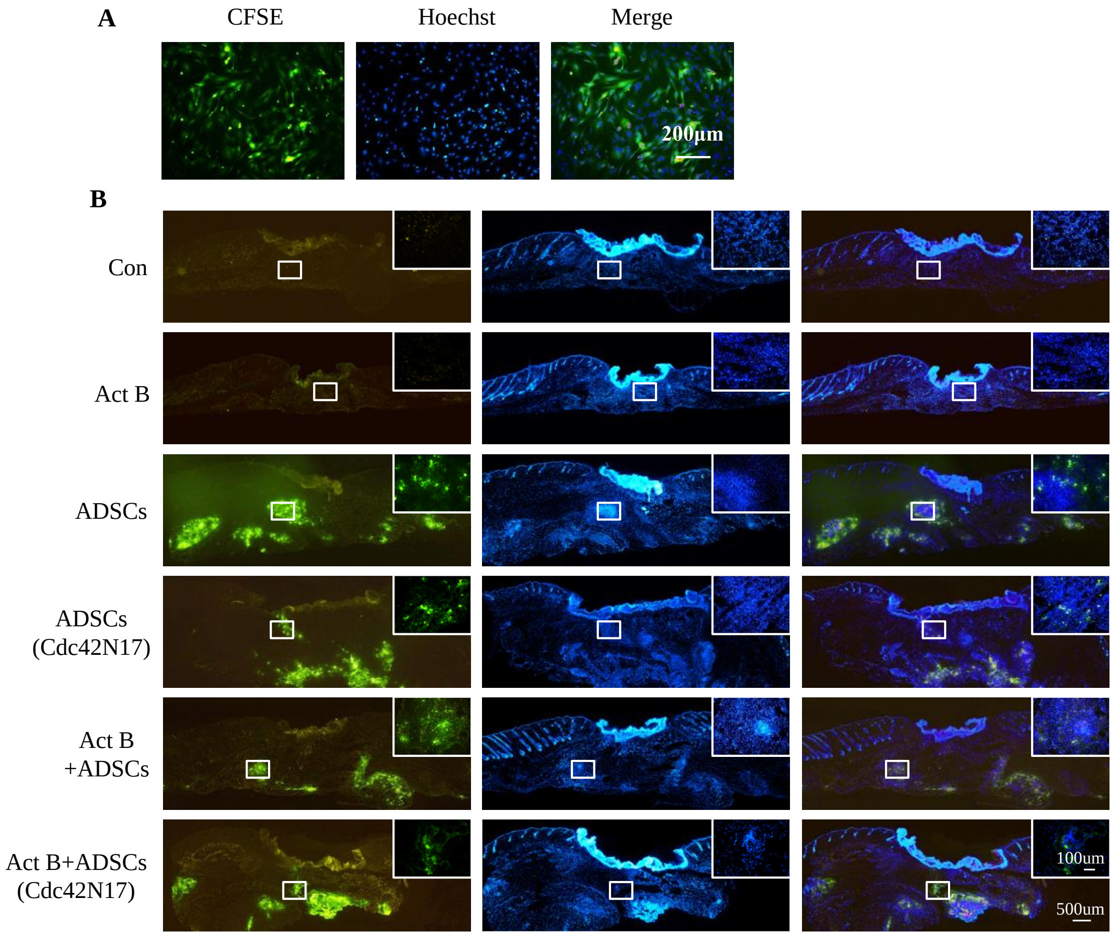

Figure.S3 ADSCs participated in cutaneous wound healing. A 5, 6-carboxyfluorescein diacetate succinimidyl ester (CFSE) was used to label ADSCs before transplantation. **B** CFSE-labeled ADSCs and Cdc42N17 transduced ADSCs were administered to the wound sites in mice of different treatment groups for 3 consecutive days. On day 3 after administration, CFSE-labeled ADSCs and Cdc42N17 transduced ADSCs were observed examined by fluorescence microscopy of frozen sections.

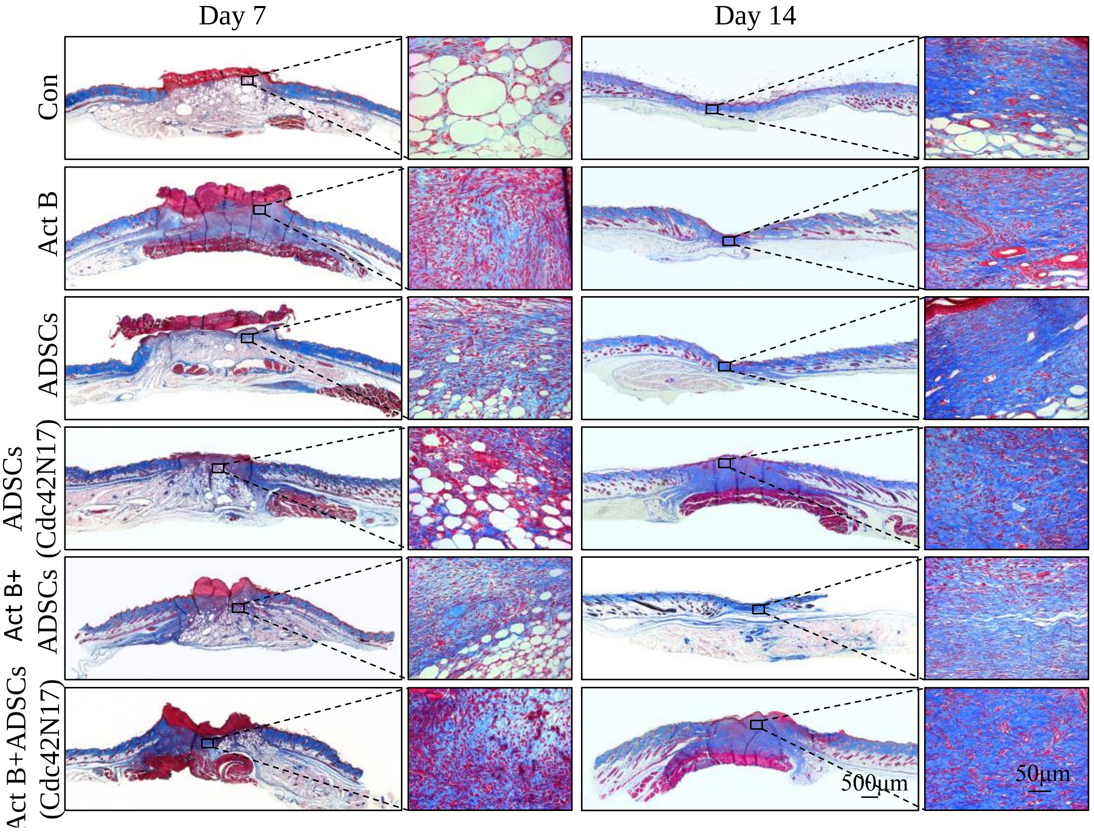

Figure.S4 Cdc42 regulates activin B-induced collagen deposition and maturation after wounding. Representative photomicrographs of Masson's trichrome-stained wounds on days 7 and 14 after wounding.

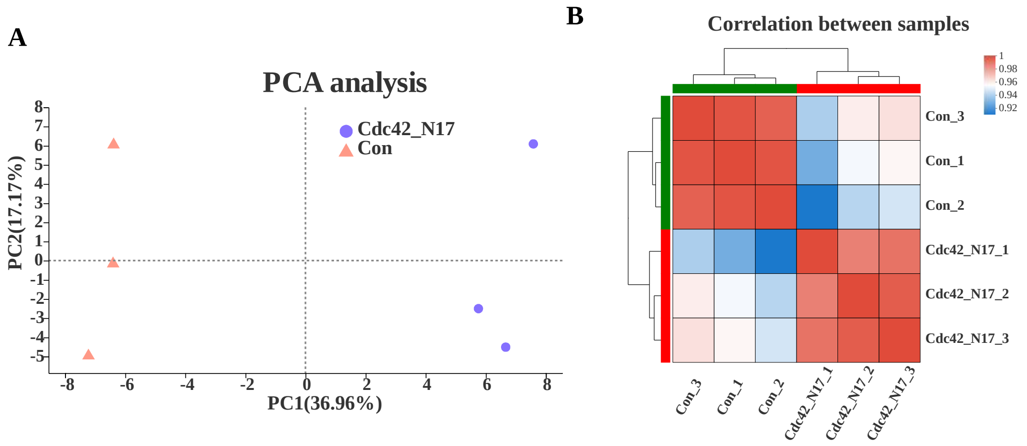

Figure.S5 Gene expression patterns analysis between the two groups of cells. **A** Principal component analysis (PCA) and **B** correlation analysis on genes expression of ADSCs and ADSCs (Cdc42N17).

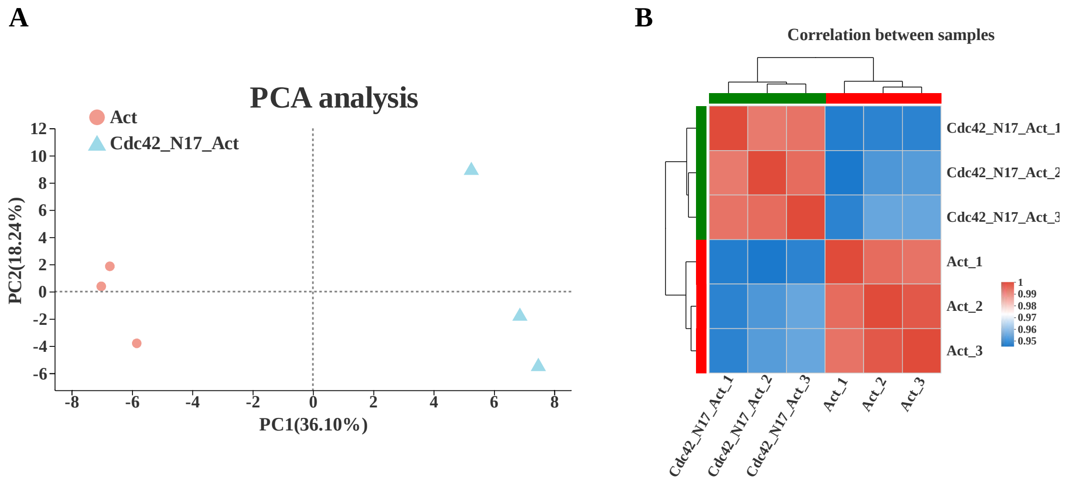

Figure.S6 Gene expression patterns analysis between the two groups of cells. **A** Principal component analysis (PCA) and **B** correlation analysis on genes expression of activin B+ ADSCs and activin B+ADSCs (Cdc42N17).
